# A new improvement: subperiosteal cocktail application to effectively reduce pain and blood loss after total knee arthroplasty

**DOI:** 10.1186/s13018-020-1563-5

**Published:** 2020-01-30

**Authors:** Yanxin Wang, Aiguo Zhou

**Affiliations:** Department of Orthopaedics, The First Affiliated Hospital of Chongqing Medical University, No.1 Youyi Road, Yuzhong District, Chongqing, 400016 China

**Keywords:** Cocktail injection, Periarticular, Subperiosteal, Pain, Blood loss, Total knee arthroplasty

## Abstract

**Background:**

Pain and blood loss after total knee arthroplasty (TKA) are unsolved clinical problems. Some studies reported that periarticular cocktail injection can effectively reduce pain and blood loss. However, there was no gold standard about the cocktail ingredient and injection location. More osteotomy and less soft tissue release in TKA with mild deformity; besides, plenty of nerves and blood vessels are contained in the periosteums and bone marrow. In this study, we aimed to detect the clinical results of subperiosteal cocktail application in TKA.

**Methods:**

Two groups were included according to the different injection location in our study. In group 1, cocktails were injected into the muscles, tendons, suprapatellar bursa, and subpatellar bursa surrounding knee joint. In group 2, cocktail injection was performed under the periosteum of the distal femur and proximal tibia. Our primary outcomes were visual analogue scale (VAS) and hemoglobin (Hb), and the secondary outcomes were wound healing, infection, deep vein thrombosis (DVT), operation time, and hospitalization.

**Results:**

At the first operative day, the mean (standard deviation) VAS score in a state of static was lower in group 2 compared with group 1 (0.98 ± 0.27 in group 1 and 0.86 ± 0.60 in group 2, *p* < 0.05). In the state of flexion and extension, the mean (standard deviation) VAS was 1.61 ± 0.66 in group 1 and 1.10 ± 0.57 in group 2 (*p* < 0.05). The mean (standard deviation) blood loss was higher in group 1 than in group 2 at the first postoperative day (440.19 (167.68) ml in group 1 and 333.67 (205.99) ml in group 2, *p* < 0.05). At the third day after surgery, the mean (standard deviation) blood loss was 686.44 (140.29) ml in group 1 and 609.19 (260.30) ml in group 2, and there was significant difference between these two groups (*p* < 0.05).

**Conclusions:**

We concluded that subperiosteal cocktail injection can significantly reduce pain and blood loss compared with periarticular cocktail injection after TKA.

## Introduction

Pain and blood loss after total knee arthroplasty (TKA) were still two of hot topic clinical problems. Many patients refused TKA due to the fear of postoperative pain [1]. In some studies, continuous epidural infiltration of the femoral or sciatic nerve [2] has been used to reduce pain in TKA. However, these methods have been associated with nerve injury, diminished muscle control, bleeding, and infection [2–6]. A study showed the amount of blood loss was approximately 1000–1790 ml during perioperative period [7], and the excessive loss of blood would lead to not only increased blood transfusion rate but also delayed rehabilitation for patients.

Periarticular cocktail injection was firstly used in TKA by Bianconi et al. [8]. Nowadays, cocktail injection is popular with surgeons which is injected into the tissues surrounding knee joint, such as the muscles, tendons, suprapatellar bursa, and subpatellar bursa. Some studies reported that periarticular cocktail injection can effectively reduce pain and blood loss [9–11]. Although there was no gold standard about the cocktail ingredient and injection location, a recent study showed that altering cocktail ingredients and targeting specific injection sites would be helpful to control pain after TKA [12].

The knee joint is composed of bones and the surrounding tissues. Periosteum is the outermost layer of bones, which can be divided into two layers. A layer of dense fibrous membrane can be observed covering the surface of periosteum. The internal surface of periosteum is rich in unmyelinated nerve fibers and small vessels. The small unmyelinated nerve fibers in the periosteum are sensitive to local anesthetics, and the abundant tiny blood vessels of periosteum communicated with the vascular in haversian canal and intramedullary.

More osteotomy and less soft tissue release in TKA with mild deformity. Besides, plenty of nerves and blood vessels are contained in the periosteums and bone marrow. In the present study, we assumed that specific cocktail injection under the periosteum would reduce pain and blood loss more effectively compared with periarticular cocktail injection in TKA. At present, there was no research on the clinical effects of subperiosteal cocktail injection.

## Materials and methods

### Patients

**Fig. 1:**
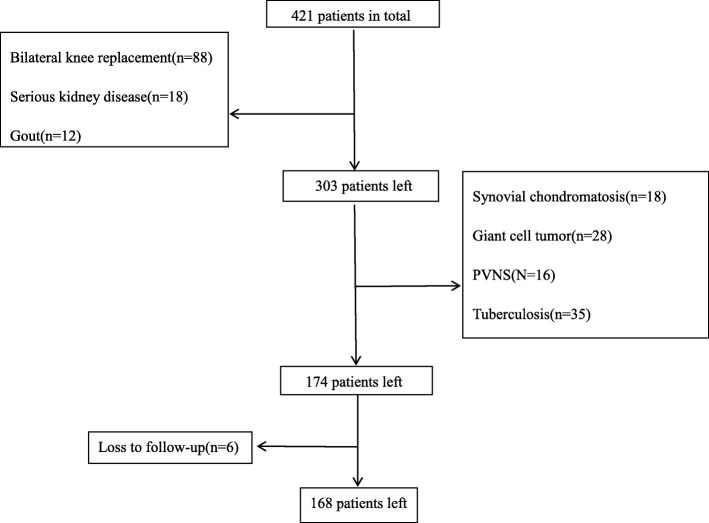
Case screening process

This was a prospective, non-randomized controlled trial, which has been approved by the ethics committee of our hospital. From 2015 to 2018, a total of 421 patients underwent osteoarthritis in our hospital and only 168 patients were chosen after screening (Fig. 1). All patients were divided into two groups; there were 82 patients in group 1 and 86 patients in group 2. The inclusion criteria were knee osteoarthritis, rheumatoid arthritis, age > 60 years old, severe deformities (> 30° flexion contracture, > 30° valgus, > 20° varus), and body mass index < 30 kg/m^2^. The exclusion criteria were bilateral knee arthroplasty, preoperative medium or severe anemia, gout, tumor, tuberculosis, pigmented villondular synovitis, arteriosclerosis occlusion, and ligament injury.

Operations were performed by two experienced osteopathic physicians. After knowing the advantages and disadvantages of various types of prosthesis, patients made their own choices.

All perioperative treatments for patients were administered under the guidance of enhanced recovery after surgery (ERAS). Patients underwent a routine blood test in the outpatient setting once they requested an operation, and they underwent treatment at outpatient if they were diagnosed with anemia (iron supplementation and erythropoietin injection). Diagnosis of anemia was based on a hemoglobin level > 11 g/dl for female or 12 g/dl for male.

### Surgical technique and postoperative care

Intravenous antibiotics were given before surgery to prevent infection, and 1.5 g of tranexamic acid (TXA) was given to reduce perioperative blood loss. During the operation, the mean arterial pressure was maintained between 50 and 65 mmHg to reduce bleeding [2] and the temperature in the operating room was tightly controlled.

**Fig. 2:**
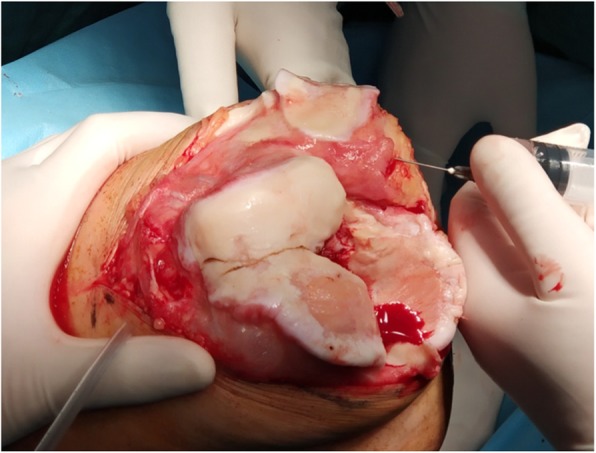
Periarticular cocktail injection. Cocktails were injected into the muscles, tendons, suprapatellar bursa, and subpatellar bursa surrounding knee joint

**Fig. 3:**
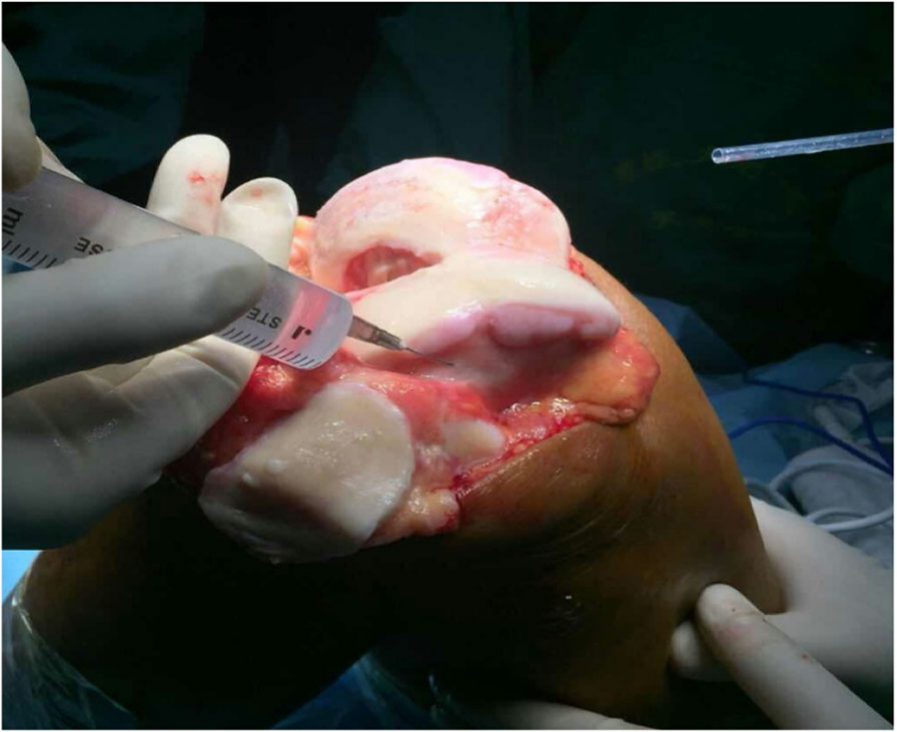
Subperiosteal cocktail injection. Cocktail injection was performed under the periosteum of the distal femur and proximal tibia

A local cocktail injection was administered before the osteotomy [13]. Cocktails were injected into the muscles, tendons, suprapatellar bursa, and subpatellar bursa surrounding knee joint in group 1 (Fig. 2). Cocktail injection was performed under the periosteum of the distal femur and proximal tibia in group 2 (Fig. 3). In the present study, TXA, epinephrine, methylprednisolone, and ropivacaine were diluted to a total volume of 100 ml with normal saline, and this cocktail ingredient has been used for many years in our hospital. TXA can remarkably decrease blood loss and mitigate reductions in hemoglobin (Hb) levels [14–18] by inhibiting fibrinolysis [19]. Epinephrine can effectively prolong the action time of the drug by contracting the anterior sphincter of arteriole and capillaries. Ropivacaine is characterized by sensory and motor block separation at low concentrations. Methylprednisolone can help in anti-inflammatory analgesic [20, 21]. Tourniquet and drainage tube were not used in surgery.

Two groups had the same anticoagulation and analgesia methods in the perioperative period. Low molecular weight heparin (LMWH) and air pressure treatment were used to prevent deep vein thrombosis (DVT) at the first postoperative day. Multiple modes combined analgesia was applied for all patients. Patients could walk with the help of the rehabilitation doctors and perform muscle functional and range-of-motion exercise at the first day after surgery.

### Outcome assessment

Age, sex, body mass index (BMI), and preoperative anemia rate were recorded in both groups.

The primary outcomes were visual analogue scale (VAS) and Hb. The pain was assessed by VAS score at the first and third postoperative day, and scores were recorded by the same doctor where 0 was no pain and 10 was unbearable pain. The blood was drawn at the first and third morning after surgery. The total blood loss in our study could be divided into intraoperative bleeding and hidden blood loss (HBL). HBL was mainly caused by extravasation into the tissues, residual blood in the joint, and hemolysis [22–30]. And blood loss was calculated using a mathematical formula according to Hb (grams per liter), height, sex, and weight. Patient blood volume (PBV) was calculated according to the classic method of Nadler and colleagues [31], including sex, height, and weight: For males, PBV = (366.9*h*^3^) + (32.19*w*) + 604.1 (ml); for females, PBV = (356.1*h*^3^) + (33.08*w*) + 183.3 (ml), where *h* = height in meters and *w* = weight in kilograms. Hb loss could be estimated by the following formula [27]: Hb_loss_ = BV × (Hb_i_ − Hb_e_) × 0.001 + Hb_t_, where Hb_i_ = the Hb concentration before surgery in grams per liter, Hb_e_ = the Hb concentration on the first or third day after surgery in grams per liter, and Hb_t_ = the amount of allogeneic Hb transfused in grams (one unit of red cell suspension was considered to contain 5 g/l of Hb in our hospital). Blood loss was related to the Hb (grams per liter) before surgery: Blood loss = 1000 × Hb_loss_/Hb_i_ (ml).

Our secondary outcomes were wound healing, infection, DVT, operative time, and hospitalization. Patients were followed up regularly at the 1.5 months, 3 months, 6 months, and 1 year after discharge. Wound healing parameters included redness, swelling, skin temperature, and sinus formation, and the diagnosis of infection was based on pathogenic evidence. Doppler ultrasound was used to examine the formation of DVT if limb swelling was evident after surgery.

### Statistical analysis

All data were analyzed using SPSS v. 17.0 statistical software (SPSS, Inc., Chicago, IL). Continuous data with a normal distribution and homogeneity of variance were evaluated using the independent sample *T* test; otherwise, the Mann-Whitney *U* test was used. *p* < 0.05 indicated a significant difference.

## Results

There were no significant differences about gender, age, BMI, operation time, rate of anemia, preoperative pain score, and hospitalization between our two groups (Fig. 1 and Table 1).

**Table 1:** General data

	Group 1	Group 2	*p* value
Gender (M:F)	25:61	23:59	1
Age* (years)	71.95 ± 6.75	70.08 ± 7.281	> 0.05
BMI*	23.83 ± 2.84	23.84 ± 2.86	> 0.05
Operation time* (min)	62.22 ± 8.82	60.92 ± 10.04	> 0.05
Rate of anemia	30:82	33:86	1
Hospitalization*	6.77 ± 1.39	6.53 ± 1.44	> 0.05

*The data are shown as the mean (standard deviation)

### Blood loss

At the first postoperative day, the blood loss was 440.19 (167.68) ml in group 1 and 333.67 (205.99) ml in group 2. At the third day after surgery, the blood loss was 686.44 (140.29) ml in group 1 and 609.19 (260.30) ml in group 2. Blood loss in the subperiosteal cocktail injection group was less than that in the periarticular cocktail injection group (*p* < 0.05). Only one patient required blood transfusion (two units of red blood cell suspension) in the periarticular cocktail injection group (Table 2), this patient complained of dizziness, and the hemoglobin was 74 g/l. It suggested that patients in the subperiosteal cocktail injection group had a better hemostatic effect and no patients in this group needed a blood transfusion. There was no difference about the decrease of Hb level in our two groups after surgery (*p* > 0.05).

**Table 2:** Hb and blood loss

	Group 1	Group 2	*p* value
Hb decline^&^ (day 1)	13.07 ± 6.38	13.07 ± 6.38	> 0.05
Hb decline^&^ (day 3)	21.54 ± 5.8	21.54 ± 5.8	> 0.05
Blood loss^&^ (day 1)	440.19 (167.68)	440.19 (167.68)	< 0.05
Blood loss^&^ (day 3)	686.44 (140.29)	686.44 (140.29)	< 0.05

*Hb* hemoglobin (g/l)

^&^The data are presented as the median (interquartile range)

### Pain score

The degree of pain after surgery was evaluated by VAS. At the first postoperative day, VAS in the state of static and in the state of flexion and extension were 0.98 ± 0.27 and 1.61 ± 0.66 in group 1, compared to 0.86 ± 0.60 and 1.10 ± 0.57 in group 2. The level of pain in subperiosteal cocktail injection group was significantly decreased compared with that in periarticular cocktail injection group (*p* < 0.05). However, there was no difference about the pain score between the two groups at the third day after surgery (*p* > 0.05) (Table 3). These results might be related to the efficacy of cocktails and the interference from other painkillers.

**Table 3:** VAS

	Group 1	Group 2	*p* value
Day^A^ 1	0.98 ± 0.27	0.86 ± 0.60	< 0.05
Day^B^ 1	1.61 ± 0.66	1.10 ± 0.57	< 0.05
Day^A^ 3	1.17 ± 0.38	1.02 ± 0.51	> 0.05
Day^B^ 3	1.98 ± 0.70	1.55 ± 0.50	> 0.05
Day^C^ 3	0.95 ± 0.47	0.10 ± 0.30	> 0.05

^A^VAS score in a static state

^B^VAS score in the flexion state of the joint

^C^VAS score while walking on flat ground

No patient was diagnosed with wound infection in our two groups. 12.2% of the patients in group 1 and 9.3% of the patients in group 2 experienced DVT in the lower extremity, which meant that subperiosteal cocktail injection would not increase the rate of wound infection and DVT in TKA while reducing the blood loss and pain.

## Discussion

Pain and blood loss after TKA were two focused issues, which were related to patient’s fear, increased blood transfusion rate, and delayed rehabilitation [1, 7]. Many studies have reported that periarticular cocktail injection would effectively reduce pain and blood loss [9–11]. In this method, cocktails were injected into the muscles, tendons, suprapatellar bursa, and subpatellar bursa surrounding knee joints to have an effect on the nerves and blood vessels. However, a recent study showed that altering cocktail ingredients and targeting specific injection sites would be helpful to control pain after TKA [12].

There was no gold standard about the cocktail ingredient and injection location.

In our new method, cocktails were composed of TXA, epinephrine, methylprednisolone, and ropivacaine which were injected under the periosteum. We supposed that subperiosteal cocktail injection would bring better analgesic and hemostatic effects after TKA, and summarized the following reasons: Plenty of nerves and blood vessels are contained in the periosteum and bone marrow. Dense fibrous membrane is observed covering the surface of periosteums, which may reduce the effects of cocktail. In this study, we compared the analgesia and hemostatic efforts of subperiosteal cocktail injection and periarticular cocktail injection.

In the present study, patients in the subperiosteal cocktail injection group felt less pain at the first day after TKA. This result was in line with our assumption above [32]. Cocktails could directly act on the subperiosteal nerves and might bring better analgesia effects. However, similar pain level was not found at the third postoperative day, and we also summarized the reasons for this result: Firstly, the efficacy of cocktails might be significantly decreased or disappeared at the third day after surgery. Secondly, the same multimodal analgesia scheme was administered for patients after TKA, and the effect of the cocktail might be obscured after the painkillers took effect.

The same anticoagulant and hemostatic treatments were given in our two groups after TKA. However, less blood loss was observed in the subperiosteal cocktail injection group, which meant that this method was a more effective way to reduce blood loss compared with periarticular cocktail injection. There was no significant difference in anemic rate before surgery, and only one patient in the periarticular cocktail injection group received blood transfusion after surgery; also, the rates of wound infection and DVT were not increased in the subperiosteal cocktail injection group, which meant that this technique could reduce blood loss without increasing complications [33, 34]. Otherwise, the reduced blood loss would also be helpful to decrease the prosthesis loosening rate [35–37].

There were some limitations in our study: A large sample and long-term follow-up are needed to evaluate the clinical efforts of these two techniques. The molecular mechanism analysis is needed to further improve the clinical effect. The blank control is needed to precisely detect the clinical effect of these two groups.

## Conclusion

This was the first time to study the clinical effects of subperiosteal cocktail injection. We concluded that subperiosteal cocktail injection was an effective method to reduce pain and blood loss in the early period after TKA and did not increase the rate of wound infection and DVT. Subperiosteal cocktail injection might be a better method to improve the prognosis of TKA.

## Data Availability

The raw data are available from the corresponding author on reasonable request.

## Abbreviations

ASO: Arteriosclerosis occlusion
BMI: Body mass index
DVT: Deep vein thrombosis
ERAS: Enhanced recovery after surgery
F: Female
Hb: Hemoglobin
HBL: Hidden blood loss
LMWH: Low molecular weight heparin
M: Male
PBV: Patient blood volume
PVNS: Pigmented villonodular synovitis
TKA: Total knee arthroplasty
TXA: Tranexamic acid
VAS: Visual analogue scale
